# Cerebral Mitochondrial Function and Cognitive Performance during Aging: A Longitudinal Study in NMRI Mice

**DOI:** 10.1155/2020/4060769

**Published:** 2020-04-13

**Authors:** Martina Reutzel, Rekha Grewal, Benjamin Dilberger, Carmina Silaidos, Aljoscha Joppe, Gunter P. Eckert

**Affiliations:** ^1^Institute of Nutritional Sciences, Laboratory for Nutrition in Prevention and Therapy, Justus-Liebig-University of Giessen, Biomedical Research Center Seltersberg (BFS), Schubertstrasse 81, 35392 Giessen, Germany; ^2^Department of Biological Sciences & Cluster of Excellence Macromolecular Complexes, Institute of Molecular Biosciences, Johann Wolfgang Goethe University Frankfurt, Frankfurt am Main, Germany

## Abstract

Brain aging is one of the major risk factors for the development of several neurodegenerative diseases. Therefore, mitochondrial dysfunction plays an important role in processes of both, brain aging and neurodegeneration. Aged mice including NMRI mice are established model organisms to study physiological and molecular mechanisms of brain aging. However, longitudinal data evaluated in one cohort are rare but are important to understand the aging process of the brain throughout life, especially since pathological changes early in life might pave the way to neurodegeneration in advanced age. To assess the longitudinal course of brain aging, we used a cohort of female NMRI mice and measured brain mitochondrial function, cognitive performance, and molecular markers every 6 months until mice reached the age of 24 months. Furthermore, we measured citrate synthase activity and respiration of isolated brain mitochondria. Mice at the age of three months served as young controls. At six months of age, mitochondria-related genes (complex IV, creb-1, *β*-AMPK, and Tfam) were significantly elevated. Brain ATP levels were significantly reduced at an age of 18 months while mitochondria respiration was already reduced in middle-aged mice which is in accordance with the monitored impairments in cognitive tests. mRNA expression of genes involved in mitochondrial biogenesis (cAMP response element-binding protein 1 (creb-1), peroxisome proliferator-activated receptor gamma coactivator 1-alpha (PGC1-*α*), nuclear respiratory factor-1 (Nrf-1), mitochondrial transcription factor A (Tfam), growth-associated protein 43 (GAP43), and synaptophysin 1 (SYP1)) and the antioxidative defense system (catalase (Cat) and superoxide dismutase 2 (SOD2)) was measured and showed significantly decreased expression patterns in the brain starting at an age of 18 months. BDNF expression reached, a maximum after 6 months. On the basis of longitudinal data, our results demonstrate a close connection between the age-related decline of cognitive performance, energy metabolism, and mitochondrial biogenesis during the physiological brain aging process.

## 1. Introduction

The average life expectancy has increased considerably to over 80 years in developed countries [1], and the multifactorial aging process is characterized by several changes on the cellular level [2, 3]. Mitochondria are cell organelles with central functions such as energy metabolism, including ATP production and generation of reactive oxygen species (ROS); however, mitochondrial dysfunction has been identified as an important hallmark of aging [4–9]. In addition, there are many studies that describe the close relationship between various age-related diseases and impaired mitochondrial function, which makes mitochondria interesting as a potential target for the treatment and prevention of neurodegenerative diseases [10, 11]. Mitochondrial dysfunction is characterized by a reduced efficiency of the respiratory chain system diminishing the synthesis of high-energy molecules such as ATP and the expression of genes involved in mitochondrial biogenesis, cellular longevity, and the antioxidant defense systems [12]. Evidences point out that the activity of complex I and complex IV of the respiratory chain system is impaired in aged brains which leads to a reduced capability to produce ATP [13, 14]. In 1956, the “free radical, theory of aging” was postulated by Harman which states that cellular aging is a direct consequence of free radicals, especially the superoxide anion radical (O_2_^·−^), attacking cells and tissue [3, 15–17]. This framework has been refined over the last years. In 1979, mitochondria were identified as the key producer of ROS that significantly contribute to aging processes [18–21]. However, low “physiological” ROS levels are known to have important functions for signaling mechanisms in the cell [22]. Under physiological conditions, the antioxidative defense system, including superoxide dismutase (SOD) and the glutathione (GSH) system, is able to eliminate highly reactive molecules [23]. However, if there is an imbalance between the generation of ROS and the cellular defense system, oxidative damage occurs which can initiate apoptosis and trigger neurodegenerative diseases. Furthermore, aging is characterized by changes in mitochondrial dynamics [24]: these organelles are able to fuse and to divide. The later process, the so-called fission, is part of the cellular quality control and results in fragments of different sizes that are cleared by mitophagy [25, 26]. The fission processes also help to regulate the cellular ATP levels. Fusion leads to enrichment of mtDNA and finally reduces mutations, [24].

Most of the aging studies in rodents conducted so far compared aged animals to young ones but did not collect longitudinal data over the entire lifetime. Thus, studies on cognitive performance and bioenergetic parameters in the brain covering the lifespan are rare. Therefore, we measured the development of the energy metabolism and mRNA expression of genes involved in mitochondrial biogenesis, antioxidant capacity, and synaptic plasticity in the brain as well as the cognitive performance every six months in the same cohort of female NMRI mice. Female NMRI mice are a well-described outbred mouse model for the physiological, “normal” aging process which reflects a high variability of the genome [27–29]. This model has been described as most suitable for studies on physiological aging compared to inbred or genetically modified mouse models with accelerated aging or reduced lifespans [30, 31].

## 2. Material and Methods

### 2.1. Animals and Treatment

Female NMRI mice (Navar Medical Research Institute) were purchased at the age of 3 weeks from Charles River (Sulzbach, Germany) and kept in the animal station until they reached the ages of 3, 6, 12, 18, and 24 months. All mice had ad libitum access to a standard pelleted diet (cat. no.1324; Altromin, Lage, Germany) and drinking water. Behavioral testing was performed before all time points. Mice were sacrificed by decapitation. The brain was quickly dissected on ice after the removal of the cerebellum, the brain stem, and the olfactory bulb. All experiments were carried out by individuals with appropriate training and experience according to the requirements of the Federation of European Laboratory Animal Science Associations and the European Communities Council Directive (Directive 2010/63/EU). Experiments were approved by the regional authority (Regierungspraesidium Darmstadt; #V54–19 c 20/15–FU/1062).

### 2.2. Passive Avoidance Test

The test was carried out using a passive avoidance step-through system (cat. no. 40533/mice; Ugo Basile, Germonio, Italy) and a protocol similar to the protocol published by Shiga et al. [32]. On day one of the experiment, the mouse was put into the light chamber (light intensity 75%). The door toward the dark chamber was opened after a 30 s delay, and time was recorded until the mouse enters into the dark chamber. In the dark chamber, the mouse received an electric shock (0.5 mA, 1 s duration). If the mouse did not enter the dark chamber after 180 s, the test was stopped. The same test was repeated 24 h later. This time, the door toward the dark chamber was already opened after 5 s and time was measured until the mouse entered the dark chamber but the electric shock was turned off. The test was aborted after 300 s.

### 2.3. One-Trial Y-Maze Test

A one-trial Y-maze test was conducted using a custom-made Y-maze (material: polyvinyl chloride; length of arms: 36 cm; height of arms: 7 cm; width of arms: 5 cm; and angle between arms: 120°). At the beginning of the test, the mouse was put into one of the three arms of the Y-maze, and the sequence of the entries was recorded for 5 min. Spontaneous alternation was determined using the formula (number of alternations/number of entries)/2 [33].

### 2.4. Preparation of Dissociated Brain Cells

One hemisphere of the brain was used to prepare dissociated brain cells (DBCs) for ex vivo studies. The brain was washed once in medium 1 (138 mM NaCl, 5.4 mM KCl, 0.17 mM Na_2_HPO_4_, 0.22 mM KH_2_PO_4_, 5.5 mM glucose∗H_2_O, and 58.4 mM sucrose; pH = 7.35). Afterwards, it was cut into small pieces in 2 ml of medium 1 using a scalpel. The chopped brain was then pressed through a 200 *μ*m nylon mesh into a beaker containing 16 ml of medium 1 using a plastic Pasteur pipette with a wide opening. In the last step, the brain homogenate was filtered through a 102 *μ*m nylon mesh. The resulting brain homogenate was centrifuged (2000 rpm, 5 min, and 4°C) before the pellet was redissolved in 20 ml of medium 2 (110 mM NaCl, 5.3 mM KCl, 1.8 mM CaCl_2_∗2 H_2_O, 1 mM MgCl_2_∗6 H_2_O, 25 mM Glucose∗H_2_O, 70 mM sucrose, and 20 mM HEPES). The centrifugation step was repeated twice; after the last centrifugation, the pellet was redissolved in 4.5 ml of Dulbecco's modified without supplements. DBCs were seeded in 250 *μ*l aliquots in 12 replicates into a 24-well plate for the measurement of the mitochondrial membrane potential. For the measurement of the ATP level, DBCs were seeded in 50 *μ*l aliquots into a 96-well plate. Cells were incubated for 3 h in a humidified incubator (5% CO_2_). Respectively, 6 wells were incubated for 3 h with sodium nitroprusside (0.5 mM for ATP measurement; 2 mM for the measurement of the mitochondrial membrane potential) in DMEM. The remaining cell suspension was reserved for protein determination and stored at -80°C.

### 2.5. Measurement of ATP Concentrations in DBCs

The ViaLight Plus bioluminescence kit (Lonza, Walkersville, USA) was used for assessing ATP concentrations in DBC. At the end of the incubation, the 96-well plate was removed from the incubator and allowed to cool to room temperature for 10 min. Afterwards, all wells were incubated with 25 *μ*l lysis buffer in the dark for 10 min. In the next step, wells were incubated with 50 *μ*l monitoring reagent. The emitted light (bioluminescence) was recorded using a luminometer (Victor X3 multilabel counter). The ATP concentrations in the wells were determined using a standard curve; ATP concentrations of DBC were normalized to protein content.

### 2.6. Measurement of Mitochondrial Membrane Potential

MMP was measured in DBC using the fluorescence dye Rhodamine123 (R123). DBCs were incubated in an incubator (37°C, 5% CO_2_) for 15 min with 0.4 *μ*M R123. Afterwards, the reaction was stopped by adding Hank's Balanced Salt Solution (HBSS) into the wells. DBCs were centrifuged (914 g, 5 min, room temperature), the medium was aspirated, and DBCs were supplemented with new HBSS. DBCs were triturated to obtain a homogenous sample. Subsequently, MMP was assessed by reading the R123 fluorescence at an excitation wavelength of 490 nm and an emission wavelength of 535 nm (Victor X3 multilabel counter). The fluorescence in each well was read in four consecutive runs. The fluorescence values were then normalized to protein content.

### 2.7. Isolation of Brain Mitochondria and High-Resolution Respirometry

Half a brain hemisphere (the frontal part) was used to isolate brain mitochondria. The protocol is described in Hagl et al. [34]. The pellet obtained from the last centrifugation step was dissolved in 250 *μ*l MIRO5 (0.5 mM EGTA, 3 mM MgCl_2_∗6 H_2_O, 60 mM K-lactobionat, 20 mM taurine, 10 mM KH_2_PO_4_, 20 mM HEPES, 100 mM sucrose, 1 g/l BSA). Subsequently, 80 *μ*l of the resulting cell suspension was injected into an Oxygraph 2k-chamber. A complex protocol was used to investigate the function of the respiratory chain complexes. The capacity of the oxidative phosphorylation (OXPHOS) was determined using complex I-related substrates pyruvate (5 mM), malate (2 mM), and ADP (2 mM) followed by the addition of succinate (10 mM). Mitochondrial integrity was measured by addition of cytochrome c (10 *μ*M). Oligomycin (2 *μ*g/ml) was added to determine leak respiration (leak (omy)), and afterwards, uncoupling was achieved by carbonyl cyanide p-(trifluoromethoxy)phenylhydrazone (FCCP, injected stepwise up to 1-1.5 *μ*M). Complex II respiration was measured after the addition of rotenone (0.5 *μ*M). Complex III inhibition was achieved by the addition of antimycin A (2.5 *μ*M) and was subtracted from all respiratory parameters. COX activity was measured after ROX determination by applying 0.5 mM tetramethylphenylenediamine (TMPD) as an artificial substrate of complex IV and 2 mM ascorbate to keep TMPD in the reduced state. Autoxidation rate was determined after the addition of sodium azide (>100 mM), and COX respiration was additionally corrected for autoxidation.

### 2.8. Citrate Synthase Activity

Citrate synthase activity was measured photometrically in isolated brain mitochondria as described in Hagl et al. [34].

### 2.9. Protein Quantification

Protein content was determined according to the BCA method using a Pierce™ Protein Assay Kit (Fisher Scientific, Waltham, MA, USA) according to the manufacturer's instructions.

### 2.10. Transcription Analysis by Quantitative Real-Time PCR (qRT-PCR)

Total RNA was isolated using the RNeasy Mini Kit (Qiagen, Hilden, Germany) according to the manufacturer's instructions using ~20 mg RNAlater stabilized samples (Qiagen, Hilden, Germany). RNA was quantified measuring the absorbance at 260 and 280 nm using a NanoDrop™ 2000c spectrometer (Thermo Fisher Scientific, Waltham, MA, USA). RNA purity was assessed using the ratio of absorbance 260/280 and 260/230. To remove residual genomic DNA, samples were treated with a TURBO DNA-free™ kit according to the manufacturer's instructions (Thermo Fisher Scientific, Waltham, MA, USA). Complementary DNA was synthesized from 250 ng total RNA using the iScript cDNA Synthesis Kit (BioRad, Munich, Germany) according to the manufacturer's instructions and was stored at -80°C. qRT-PCR was conducted using a CfX 96 Connect™ system (BioRad, Munich Germany). Oligonucleotide primer sequences, primer concentrations, and product sizes are listed in Table 1. All primers were received from Biomol (Hamburg, Germany) or Biomers (Ulm, Germany). cDNA for qRT-PCR was diluted 1 : 5 with RNase-free water (Qiagen, Hilden, Germany), and all samples were performed in triplicates. PCR cycling conditions were an initial denaturation at 95°C for 3 min, followed by 45 cycles of 95°C for 10 s, 58°C for 45 s, and 72°C for 29 s. Gene expression was analyzed using the −(2*ΔΔ*C_q_) method using BioRad CfX manager software and was normalized to the expression levels of beta 2 microglobulin (B2M) and phosphoglycerate kinase 1 (PGK1).

### 2.11. Statistics

Unless otherwise stated, values are presented as mean ± standard error of the mean (SEM). Statistical analyses were performed by applying one-way ANOVA with Tukey's multiple comparison post-test (Prism 8.0 GraphPad Software, San Diego, CA, USA). Statistical significance was defined for *p* values of < 0.05.

## 3. Results

Female NMRI mice of one cohort were investigated at 3, 6, 12, 18, and 24 months of age. Mice aged 24 months had to be excluded from both of the cognitive tests, since they were too impaired in their mobility.

### 3.1. Effect of Aging on Cognitive Performance

Spatial learning memory and locomotor activity were determined using the Y-maze spontaneous alternation test in 3-, 6-, 12-, and 18-month-old mice [29, 35, 36].

**Figure 1:**
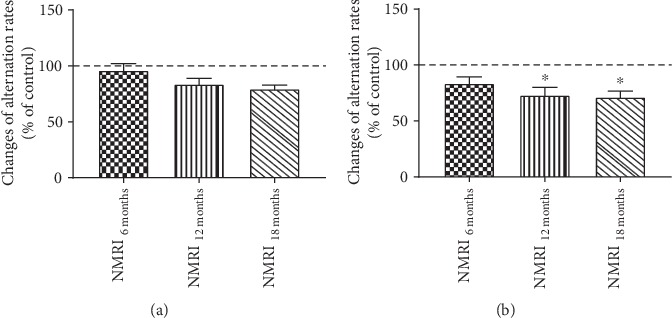
Y-Maze spontaneous alternation test of 3-, 6-, 12-, and 18-month-old mice during a five-minute period time of testing. Changes of alternation rates (% of control) (a) and changes of number of alternations (% of control) (b); *n* = 12, mean ± SEM, and one-way ANOVA with Tukey's post hoc test; ^∗^*p* < 0.05. Performance of young control mice (3 months) is defined as 100%.

Mice, aged 6 or 12 months, showed slightly but not significantly decreased changes in alternation rates compared to young controls during a five-minute testing phase (Figure 1(a)). Significant changes were observed at an age of 18 months (-21%) (Figure 1(a)). Regarding the number of alternations, 6- and 12-month-old NMRI mice showed numerically but not significantly reduced performance compared to young animals (Figure 1(b)). Significantly reduced changes in the number of alternations were recorded starting at an age of 12 months (-28%) (Figure 1(b)).

**Figure 2:**
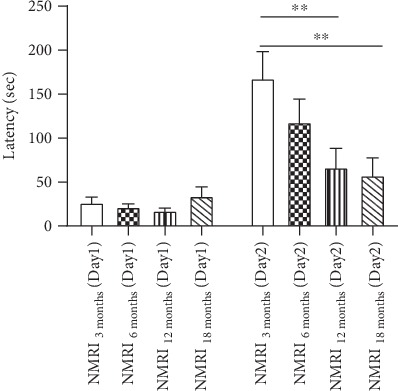
Passive avoidance test with 3-, 6-, 12-, and 18-month-old NMRI mice. On day one, mice receive a mild electric shock (0.5 mA) and time that the mouse needs to enter into the dark chamber is recorded; 24 h after the first testing period, the test is repeated and time that the mouse needs to reenter the dark chamber is recorded; *n* = 15, mean ± SEM, and one-way ANOVA with Tukey's post hoc test; ^∗^*p* < 0.05 and ^∗∗^*p* < 0.01.

The fear-based passive avoidance test was used to assess the cognitive performance in 3-, 6-, 12-, and 18-month-old mice [37, 38]. Young mice remembered the foot shock they received when they entered the dark chamber 24 hours before quite well as indicted by an increased latency time on day two (Figure 2). On day two, old animals reentered the dark chamber faster than young control animals resulting in significantly lower latency times (Figure 2) indicating a lack in memory in mice aged 12 (-60%) and 18 months (-65%). At the age of 24 months, mice showed severe impairments in mobility. Thus, they were no longer usable for the test and had to be excluded.

### 3.2. Effect of Aging on Gene Expression

**Figure 3:**
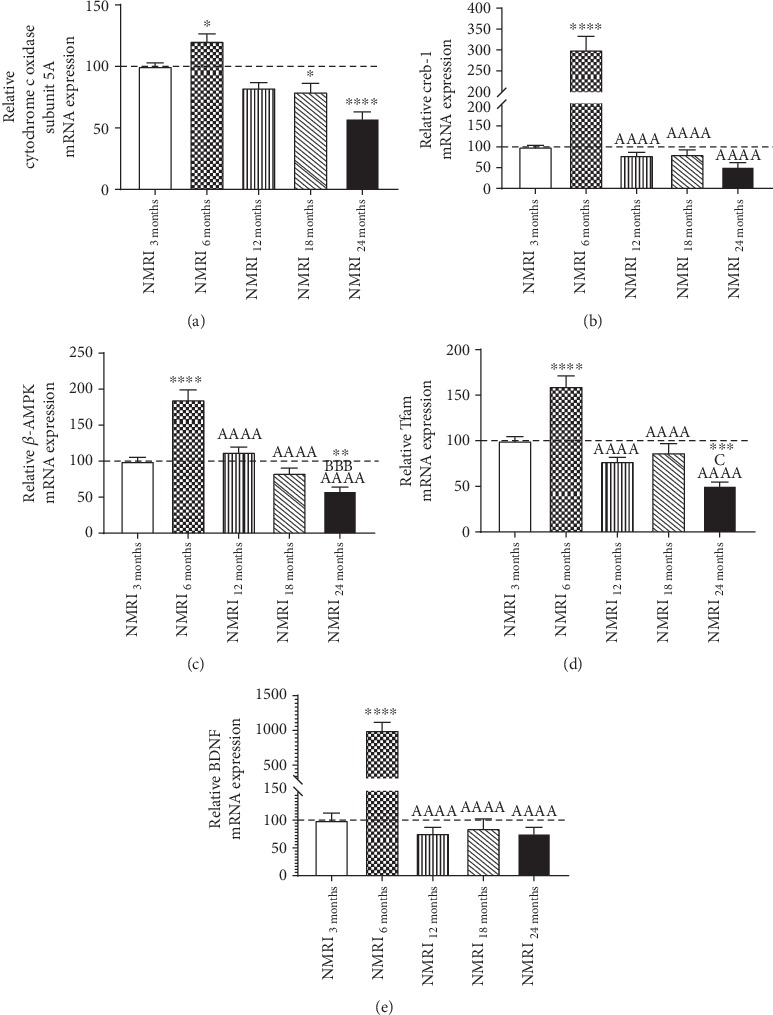
Relative normalized mRNA expression levels of cytochrome c oxidase subunit 5A (a), cAMP response element-binding protein (creb-1) (b), AMP-activated protein kinase (*β*-AMPK) (c), mitochondrial transcription factor A (Tfam) (d), and brain-derived neurotrophic factor (BDNF) (e) in brain homogenate of 3-, 6-, 12-, 18-, and 24-month-old mice. mRNA expression of 3-month-old control mice is 100%. *n* = 9, mean ± SEM with one-way ANOVA and Tukey's post hoc test with ^∗^*p* < 0.05, ^∗∗^*p* < 0.01, ^∗∗∗^*p* < 0.001, and ^∗∗∗∗^*p* < 0.0001 against 3-month-old control animals. “A” indicates one-way ANOVA and Tukey's post hoc test against 6-month-old mice, “B” against 12-month-old mice, and “C” against 18-month-old animals. Results are normalized to the mRNA expression levels of beta 2 microglobulin (B2M) and phosphoglycerate kinase 1 (PGK1).

The expression of genes involved in longevity, mitochondrial biogenesis and function, synaptic plasticity, and antioxidative properties was determined in brains of 3-, 6-, 12-, 18-, and 24-month-old mice. All genes considered showed significant changes in the expression pattern during the observed aging process in the brain. It was observed that the mRNA expression of creb-1, Tfam, complex IV, BDNF, and *β*-AMPK, which are mainly involved in mitochondrial biogenesis and physical activity, significantly increased during the young adulthood (6 months) and showed a significantly reduced expression with 24 months compared to that of young control animals (Figures 3(a)–3(e)). Furthermore, PGC1-*α* and Nrf-1, two other important transcription factors involved in mitochondrial biogenesis, showed an approximately constant expression level until the age of 24 months where mRNA expression decreased significantly in comparison to young mice (Table 2). Additionally, gene expression of the mitochondrial mass marker citrate synthase was significantly reduced starting at the age of 18 months. mRNA expression of SOD2 and Cat, enzymes responsible for the antioxidative cellular properties, significantly started to decline with 18 months. However, complex I mRNA expression was approximately constant until the age of 24 months. Expression of the synaptosomal markers SYP1 and GAP43 decreased at an age of 6 months. Unexpectedly, SYP1 mRNA expression was unchanged in 18-month-old mice only (Table 2).

### 3.3. Effect of Aging on Brain ATP Levels and Mitochondrial Membrane Potential (MMP)

ATP and MMP levels were measured in dissociated brain cells (DBCs) of 3-, 6-, 12-, 18-, and 24-month-old NMRI mice [28, 39]. ATP levels of 6- and 12-month-old mice showed no significant changes compared to those of young control animals. ATP levels were significantly reduced in DBC isolated from 18-month-old NMRI mice (-33%). However, ATP levels showed a numerical increase with 24-month-old compared to 3-month-old mice (+25%). MMP levels were significantly reduced in brains of 24-month-old NMRI mice compared to 18-month-old animals (-28%, Table 3).

### 3.4. High-Resolution Respirometry in Isolated Mitochondria

The complexes of the inner mitochondria membrane (complex I, NADH: ubiquinone oxidoreductase (CI); complex II, succinate-coenzyme Q reductase (CII); complex III, cytochrome c oxidoreductase (CIII); and complex IV, cytochrome c oxidase (CIV)) are essential for building up the mitochondrial membrane potential (MMP) which is the driving force for complex V of the mitochondrial respiration chain (F_1_/F_0_-ATPase (CV)) that produces ATP [40]. Adult mice, aged 6 months, did not show any differences concerning mitochondrial respiration compared to 3-month-old control animals (Table 3). However, mitochondrial respiration of complexes I (-30.3%) and IV (-20.4%) was significantly reduced starting at an age of 12 months compared to that of young animals (Table 3). Respiration of CI+CII (-25.6%) and CII_ETS_ (-32%) in isolated mitochondria gradually declines during aging starting at an age of 12 months (Table 4).

## 4. Discussion

In order to understand the physiological brain aging process, longitudinal aging studies are superior to single point studies since they provide a good indication of when the first cognitive deficits occurred during lifetime [41, 42]. The special characteristic of our study is that we used a single cohort of NMRI mice to examine the effects of the physiological aging process in the brain while most previous studies focused on one time point only to examine the brain aging process (Table 5). To our knowledge, we are the first who used NMRI mice of 3, 6, 12, 18, and 24 months of age to explore changes during the aging process in one cohort. In the end, these data allow a more detailed picture of mitochondrial and cognitive functions during the aging process. Overall, our data show an early decline of cognitive functions in middle-aged NMRI mice, which do not seem to go along simultaneously with the identified impairments on the molecular level and the bioenergetics of the brain. These findings are potentially important for the prevention of neurodegenerative diseases, for which aging processes are an important risk factor and can start early, well before the first symptoms appear.

One important finding in our cohort study was that mice at the age of 6 months showed a significantly different phenotype in comparison to young but also to older mice. It seems that extensive changes take place in the brain of animals at the age of 6 months, which need to be investigated in future studies. Interestingly, most aging studies use 3-month-old NMRI mice as young controls [35, 43]. Moreover, these observations demonstrate the importance of longitudinal aging studies, investigating more than one time point to describe the brain aging process and cognitive decline. Specific aspects of our study, such as study type, behavioral tests, and the role of mitochondria in brain aging, are discussed in the following sections.

### 4.1. Longitudinal Study

Longitudinal studies (Table 5, A) of the brain aging process, which provide information on mitochondrial bioenergetics in combination with cognitive function, are rare. Most longitudinal studies only consider behavioral tests or mitochondrial parameters during lifetime. More importantly, most studies did not examine mice from a cohort but reported differences at individual points in time (Table 5, B), which makes it difficult to give a clear picture of the development and course of the physiological aging process in the brain.

### 4.2. Behavioral Testing

Monitoring the following aging process, 18-month-old mice had a significantly reduced alternation rate and number of alternations compared to young control animals, indicating a reduced spatial learning memory and mobility in 18-month-old NMRI mice (Figures 1(a) and 1(b)) which is in agreement with previous studies [29, 36]. In comparison, other studies already showed a significantly decreased performance in the Y-maze test in middle-aged mice [43, 49]. Furthermore, our findings indicate that long-term memory seems to be already impaired in middle-aged mice during the physiological aging process whereas spatial learning memory seems to be more or less unaffected until the age of 18 months. The signaling molecule BDNF has several functions in brain aging and plasticity. It is an important component in biochemical pathways and is a key player in energy metabolism, neuronal survival, and neurogenesis [50–52] and has been shown to play a crucial role in hippocampus-dependent learning behavior [53]. However, during the physiological aging process, we have measured a strong decrease in gene expression of this important neurotrophic factor starting at 12 months of age. During the young adulthood of mice (6 months), BDNF mRNA expression reaches a maximum and maintains at a constant level throughout aging which is in agreement with previous findings from Webster et al. who reported a significant rise in the prefrontal cortex of BNDF in young adults [54]. Furthermore, mice lacking SYP1 show significantly reduced learning behavior [55], and an enriched environment has been reported to have positive effects on the SYP1 brain level [56]. In accordance with these findings, we detected numerically decreased SYP1 mRNA expression levels during the brain aging process starting in young adulthood which is in agreement with the observed deficits on cognition. Surprisingly, SYP1 mRNA expression increased numerically at an age of 18 months compared to 12-month-old animals. Synaptophysin is reported to be a component of neurotransmitter-containing presynaptic vesicle membranes, and its increase is closely connected to an improved neurotransmission and cognitive performance [56, 57]. However, data availability and the connection between SYP1 mRNA levels in the brain are not consistent. According to this, other studies did not show any age-related changes in SYP1 brain levels [58, 59], while others reported significantly decreased SYP1 with age [60–62]. GAP43, another nervous tissue-specific protein, is mainly involved in neurite outgrowth and elongation during the neuronal development and is also regarded as a marker for neural plasticity [63–65]. In our study, GAP43 was highly expressed in brains of 3-month-old animals which is in accordance with findings from Rosskothen-Kuhl and Illing who found the highest expression of GAP43 during the early development of the nervous system [66]. Recently, we reported that mRNA levels of all three proteins involved in neuronal plasticity were significantly decreased in brains of aged NMRI mice [39], which is confirmed by our recent data. The reduced expression of those genes indicates less synaptic plasticity and neuronal remodelling in brains of aged NMRI mice which might be one possible reason for the cognitive impairments in memory and motor performance during the aging process [67, 68].

### 4.3. Mitochondrial Bioenergetics during the Physiological Aging Process

In accordance with most of the previous studies from our group, we measured significantly reduced ATP levels in dissociated brain cells isolated from brains of 18-month-old NMRI mice in comparison to young control animals. In accordance with the finding of reduced ATP levels in brains of aged NMRI mice, we found reduced mRNA expression levels of complex IV in the brain of 18-month-old mice, which is one of the most important protein complexes involved in the oxidative phosphorylation process consistent with previous studies [28, 29, 39]. A reduced complex IV expression was associated with an increase in apoptosis [69, 70]. Bowling et al. showed an age-associated progressive decline of the respiratory chain complexes I and IV in cortices of primates [71], and Petrosillo et al. measured a reduced complex I activity in brain mitochondria of 24-month-old rats [72], whereas other studies could not confirm those findings in brains of aged mice [45]. The mitochondrial membrane potential (MMP) reflects the mitochondrial functional status and is mainly produced by complex I, complex III, and complex IV that transport protons from the mitochondrial matrix into the intermembrane space [73]. However, at the age of 24 months, we detected a small, but only numerical, increase of ATP brain levels which is in contrast to the results from Afshordel et al. who reported significantly reduced brain ATP levels in 24-month-old mice [46]. Furthermore, the group of Navarro et al. did not show any significant changes of ATP levels in brains of 13- and 19-month-old CD-1 mice [44]. Thus, age-dependent changes in ATP levels seem to depend on the strain of mice that was investigated.

Regarding the mitochondrial membrane potential, we detected reduced MMP in brains of 24-month-old mice compared to young animals which is in accordance with the reduced complex I and IV mRNA expression in brains of aged mice. Based on these data, one would expect a reduced respiration of the respiratory chain complexes [74, 75]. Accordingly, in the current study, activity of respiratory chain complexes I and IV as well as CI+CII and CII_ETS_ was significantly reduced starting at 12 months of age compared to that of young animals (Tables 3 and 4) which is in accordance with previous studies of our group detecting reduced complex activity in brains of 18-month-old animals [28, 29]. Surprisingly, our longitudinal study shows that complex activity seems to be reduced already in middle-aged animals while brain ATP and MMP levels stay constant until mice reach the age of 18 and 24 months.

### 4.4. Mitochondrial Biogenesis and the Antioxidative Defense System during the Aging Process

Mitochondrial biogenesis is a process in which new mitochondria are formed from existing mitochondria and is regulated by peroxisome proliferator-activated receptor gamma (PPAR*γ*) coactivator 1-alpha (PGC1-*α*). PGC1-*α* as a master regulator of mitochondrial biogenesis is activated by AMPK-activated kinase and Sirt-1 which are the two major pathways to induce mitochondrial biogenesis. AMPK is able to phosphorylate PGC1-*α*, or it activates Sirt-1 through increasing the NAD+ level. Alternatively, the transcription factor creb-1 pathway is induced which finally leads to the generation of new mitochondria [76]. During the brain aging process, we detected a significantly reduced mRNA expression of PGC1-*α*, Nrf-1, Tfam, creb-1, AMPK, and citrate synthase (CS), which is a key enzyme in mitochondria involved in the citric acid cycle (TCA) and thus provides information on mitochondrial mass. However, we did not find reduced citrate synthase activity, which is a common marker for mitochondrial mass (data not shown). In accordance with other studies, we were able to confirm that during the aging process, mitochondrial biogenesis seems to be impaired. For example, Picca et al. described an impaired protein expression of PGC1-*α* and Nrf-1 in liver tissue of aged rats [77]. Furthermore, overexpression of Tfam is able to reverse age-dependent memory loss in mice which shows the close connection between the detected cognitive impairments and the reduced mRNA expression of genes involved in mitochondrial biogenesis found in aged mice [78]. In contrast to our previous work which described a reduced gene expression in brains of 18-month-old mice [28, 29], in this longitudinal study, we were only able to confirm a reduced mRNA expression of most of the considered genes in brains of mice aged 24 months. We therefore hypothesize that rodents can keep physiological mRNA expression until at least the age of 18 months, but the decrease detected at 24 months of age is finally a result of the ongoing senescence [75].

Antioxidant enzymes like SOD2 and Cat1 are involved in the antioxidative defense system of the cell and are able to protect macromolecules like DNA, lipids, and proteins from oxidative damage. Thus, our findings show an age-dependent decrease of SOD2 and Cat1 starting at an age of 18 months which gives evidence that damaging effects could occur more probably in the aged mouse brain. Thus, the data availability shows variable results concerning the antioxidative enzymes. Leutner et al. showed an increased SOD activity in brains of NMRI mice starting at an age of 10 months, while other studies could not find any changes of SOD or Cat in 24-month-old rats [29, 79].

In particular, mRNA gene expression is affected first in brains of very old animals. These observations suggest that SYP1 and GAP43 [80, 81], two genes involved in synaptic plasticity and synaptogenesis, may be one possible reason for the early decline in the passive avoidance test. Furthermore, we hypothesize that the brain, as an energy-demanding organ, is able to maintain stable ATP levels until mice reach the age of 18 months, although the oxidative phosphorylation is already affected in middle-aged animals in our study. These observations suggest that glycolysis and its metabolites should be further investigated to determine the exact mechanisms behind these results since several studies showed deficits in glucose metabolism in brains of aged rats as well as healthy, old people [82, 83]. Any changes in the physiological brain glucose metabolism, mainly supported by mitochondria, affect neuronal function, cognition, learning, and memory [84].

## 5. Conclusion

Many clinical and neuropathological symptoms of AD occur parallel to the normal aging process which makes it difficult to keep them apart from each other. During the physiological aging process, several changes on cognitive performance, mitochondrial brain energy metabolism, and mRNA expression of genes involved in mitochondrial biogenesis were detected in a longitudinal study over 24 months. Most of the impairments on cognition and mitochondria bioenergetics were detected starting at an age of 18 months which in fact shows that aged NMRI mice are an appropriate model to study the physiological (brain-) aging process.

## Figures and Tables

**Table 1:** Oligonucleotide primer sequences, product sizes, and primer concentrations for quantitative real-time PCR.

Primer	Sequence	Product size (bp)	Conc. (*μ*M)
AMPK (*β*-subunit)	5′-agtatcacggtggttgctgt-3′ 5′-caaatactgtgcctgcctct-3′	190	0.1

B2M	5′-ggcctgtatgctatccagaa-3′ 5′-gaaagaccagtccttgctga-3′	198	0.4

BDNF	5′-gatgccagttgctttgtctt-3′ 5′-atgtgagaagttcggctttg-3′	137	0.1

CI (NADH-ubiquinone oxidoreductase 51 kDa subunit)	5′-acctgtaaggaccgagaga-3′ 5′-gcaccacaaacacatcaaaa-3′	227	0.1

CIV (cytochrome c oxidase subunit 5A)	5′-ctgttccattcgctgctatt-3′ 5′-gcgaacagcactagcaaaat-3′	217	0.1

Creb-1	5′-tagctgtgacttggcattca-3′ 5′-ttgttctgtttgggacctgt-3′	184	0.5

CS	5′-aacaagccagacattgatgc-3′ 5′-atgaggtcctgctttgtcct-3′	184	0.1

GAP43	5′-agggagatggctctgctact-3′ 5′-gaggacggggagttatcagt-3′	190	0.15

Nrf-1	5′-tcggagcacttactggagtc-3′ 5′-ctagaaaacgctgccatgat-3′	228	0.5

PGC1-*α*	5′-tgtcaccaccgaaatcct-3′ 5′-cctggggaccttgatctt-3′	124	0.05

PGK1	5′-gcagattgtttggaatggtc-3′ 5′-tgctcacatggctgacttta-3′	185	0.4

SOD2	5′-acagcgcatactctgtgtga-3′ 5′-gggggaacaactcaactttt-3′	183	0.1

SYP1	5′-tttgtggttgttgagttcct-3′ 5′-gcatttcctccccaaagtat-3′	204	0.1

Tfam	5′-agccaggtccagctcactaa-3′ 5′-aaacccaagaaagcatgtgg-3′	166	0.5

bp: base pairs; conc.: concentration.

**Table 2:** Relative normalized mRNA expression levels in brain homogenate from 3-, 6-, 12-, 18-, and 24-month-old mice determined using quantitative real-time PCR in comparison to 3-month-old control animals. mRNA expression of 3-month-old control mice is 100%; *n* = 9; mean ± SEM with one-way ANOVA and Tukey's post hoc test with ^∗^*p* < 0.05, ^∗∗^*p* < 0.01, ^∗∗∗^*p* < 0.001, and ^∗∗∗∗^*p* < 0.0001. “A” indicates one-way ANOVA and Tukey's post hoc test against 6-month-old mice, “B” against 12-month-old mice, and “C” against 18-month-old animals. Results are normalized to the mRNA expression levels of beta 2 microglobulin (B2M) and phosphoglycerate kinase 1 (PGK1).

Gene	6 months	12 months	18 months	24 months
Complex I (CI)	95.0 ± 3.3	102.1 ± 6.8	89.3 ± 7.0	60.3±5.4^∗∗∗^^/AA/BBB/CC^
Citrate synthase (CS)	83.1 ± 4.7	86.4 ± 7.7	69.9 ± 6.0^∗^	50.5±5.1^∗∗∗^^/A/B^
Growth-associated protein (GAP43)	66.5±2.8^∗∗∗∗^	66.6±4.8^∗∗∗∗^	80.2 ± 4.8^∗^	58.7±4.6^∗∗∗∗^^/C^
Synaptophysin 1 (SYP1)	81.4 ± 4.4^∗^	86.6 ± 2.4	103.8 ± 6.0^AA^	66.9±4.8^∗∗∗∗^^/B/CCCC^
Superoxide dismutase 2 (SOD2)	109.0 ± 5.3	81.6 ± 8.2^A^	72.7 ± 6.9^∗^^/AAA^	50.1±4.6^∗∗∗∗^^/AAAA/B^
Catalase (Cat1)	104.5 ± 3.7	89.5 ± 5.4	66.4±6.3^∗∗∗∗^^/AAAA/B^	64.2±6.8^∗∗∗∗^^/AAAA/B^
Peroxisome proliferator-activated receptor gamma coactivator 1-alpha (PGC1-*α*)	83.3 ± 3.9	82.7 ± 5.9	88.3 ± 8.5	62.5 ± 9.9^∗^
Nuclear respiratory factor-1 (NRF-1)	93.9 ± 7.8	85.7 ± 6.9	86.8 ± 9.6	51.2±4.8^∗∗∗∗^^/AA/B/CC^

**Table 3:** Basal ATP and MMP levels of dissociated brain cells (DBCs) as well as protein-normalized respiration of complexes I and IV in isolated mitochondria from 3-, 12-, 18-, and 24-month-old mice; *n* = 10; mean ± SEM; with one-way ANOVA and Tukey's post hoc test with ^∗^*p* < 0.05, ^∗∗^*p* < 0.01, and ^∗∗∗∗^*p* < 0.0001 compared to young control animals. # indicates significant changes against 18-month-old mice with *p* < 0.05.

	ATP level (nmol/mg protein)	MMP level (AU/mg protein)	CI (pmol/(s∗mg protein))	CIV (pmol/(s∗mg protein))
3 mo	1.2 ± 0.1	79323 ± 3401	1906 ± 133	8060 ± 660
6 mo	1.4 ± 0.1	86166 ± 6272	1799 ± 131	8048 ± 495
12 mo	1 ± 0.1	83357 ± 5612	1327±84^∗∗^	6412 ± 202^∗^
18 mo	0.8 ± 0.1^∗^	104125 ± 11331	1199±131^∗∗^	5591±313^∗∗^
24 mo	1.5 ± 0.2	74770 ± 4217^#^	1232±145^∗∗^	4814±489^∗∗∗∗^

**Table 4:** Protein-normalized respiration of CI+CII and CII_ETS_ in isolated brain mitochondria from 3-, 12-, 18-, and 24-month-old mice; *n* = 10; mean ± SEM; with one-way ANOVA and Tukey's post hoc test with ^∗^*p* < 0.05, ^∗∗^*p* < 0.01, ^∗∗∗^*p* < 0.001, and ^∗∗∗∗^*p* < 0.0001 compared to young control animals.

	CI+II (pmol/(s∗mg protein))	CII_ETS_ (pmol/(s∗mg protein))
3 mo	3416 ± 240	1904 ± 140
6 mo	3222 ± 158	1827 ± 79
12 mo	2539±119^∗∗^	1292±90^∗∗^
18 mo	2149±107^∗∗∗^	1000±104^∗∗∗∗^
24 mo	1605±163^∗∗∗∗^	995±109^∗∗∗∗^

**Table 5:** Results from longitudinal studies (A) and single point studies (B) focused on the results of mitochondrial bioenergetics and cognitive functions. Unless otherwise stated, 3-month-old animals served as young controls. Arrows indicate the age at which significant effects were first observed (↑ increase, ↓ decrease, and ↔ no significant effect).

	MMP	ATP	CI	CIV	Cognitive function	Mouse strain	Lit.
(A) Longitudinal studies							
Current study	↓ (24 m)	↓ (18 m)	↓ (12 m)	↓ (12 m)	↓ (12 m)	NMRI	
Navarro et al. 2005	n.d.	↔ (13 m) ↔ (19 m)	↑ (13 m) ↓ (19 m)	↓ (13 m) ↓ (19 m)	n.d.	CD-1	[44]
Kwong et al. 2000	n.d.	n.d.	↔ (13 m) ↔ (29 m)	↔ (13 m) ↔ (29 m)	n.d.	C57Bl/6	[45]
Lamberty et al. 1990	n.d.	n.d.	n.d.	n.d.	↓ (9 m) ↓ (12 m)	NMRI	[43]
Gower et al.1993	n.d.	n.d.	n.d.	n.d.	↓ (9 m) ↓ (12 m)	NMRI	[36]
(B) Single point studies							
Reutzel et al. 2018	↔	↓ (18 m)	↔	↔	↓ (18 m)	NMRI	[28]
Hagl et al. 2016	↔	↓ (18 m)	↔	↓ (18 m)	↓ (18 m)	NMRI	[27]
Afshordel et al. 2015	n.d.	↓ (24 m)	↔ (24 m)	↔ (24 m)	n.d.	NMRI	[46]
Hagl et al. 2015	↔ (18 m)	↔	↓ (18 m)	↔ (18 m)	n.d.	NMRI	[47]
Stoll et al. 1996	n.d.	n.d.	n.d.	n.d.	↔ (12 m) ↔ (22 m)	NMRI	[48]

n.d.: not determined.

## Data Availability

The data set generated during this study is available from the corresponding author on reasonable request.
